# Association of HHV‑6 reactivation and SLC6A3 (C>T, rs40184), BDNF (C>T, rs6265), and JARID2 (G>A, rs9383046) single nucleotide polymorphisms in depression

**DOI:** 10.3892/br.2024.1869

**Published:** 2024-10-03

**Authors:** Sureewan Bumrungthai, Surachat Buddhisa, Sureewan Duangjit, Supaporn Passorn, Sasiwimon Sumala, Nattaphol Prakobkaew

**Affiliations:** 1 Division of Biopharmacy, Faculty of Pharmaceutical Sciences, Ubon Ratchathani University, Ubon Ratchathani 34190, Thailand; 2 Division of Microbiology and Parasitology, School of Medical Sciences, University of Phayao, Phayao 56000, Thailand; 3 Department of Medical Technology, Faculty of Allied Health Sciences, Burapha University, Chonburi 20131, Thailand; 4 Division of Pharmaceutical Chemistry and Technology, Faculty of Pharmaceutical Sciences, Ubon Ratchathani University, Ubon Ratchathani 34190, Thailand; 5 Division of Biotechnology, School of Agriculture and Natural resources, University of Phayao, Phayao 56000, Thailand

**Keywords:** human herpesvirus 6, solute carrier family 6 member 3, brain-derived neurotrophic factor, jumonji and AT-rich interaction domain-containing 2, single nucleotide polymorphism, major depressive disorder

## Abstract

Major depressive disorder (MDD) is a global health concern with a complex etiology involving genetic, environmental and infectious factors. The exact cause of MDD remains unknown. The present study explored the association between genetic factors, human herpesvirus 6 (HHV-6) and MDD. The present study analyzed single nucleotide polymorphisms (SNPs) and HHV-6 viral load in oral buccal samples from patients with MDD (with and without blood relatives with MDD) and healthy controls. The study used high-resolution melt analysis to examine rs40184 (C>T) in the solute carrier family 6 member 3 (SLC6A31) gene, rs6265 (C>T) in the brain-derived neurotrophic factor (BDNF) gene and rs9383046 (G>A) in the jumonji and AT-rich interaction domain-containing 2 (JARID2) gene. HHV-6 infection and viral load was assessed using the quantitative PCR. Whole-exome sequencing was used to examine SNPs. The variant alleles of SNPs rs40184 [18/40 (45.00) vs. 29/238 (12.55%)] and rs6265 [30/54 (55.46) vs. 117/292 (40.06%)] were significantly more common in patients with MDD than in healthy controls, indicating they may be probable hereditary risk factors for MDD. HHV-6 positivity was significantly more common in carriers of the G/A genotype (12/15, 80%) than carriers of the G/G genotype (75/363, 20.7%) for rs9383046, implying that genetic variations may affect HHV-6 risk and MDD onset. Similarly, HHV-6 viral loads were significantly higher in carriers of the G/A genotype (99,990.85±118,392.64 copies/ng DNA) than carriers of the G/G genotype (48,249.30±101,216.28 copies/ng DNA) for rs9383046. Whole-exome sequencing identified two SNPs in JARID2 (rs11757092 and rs9383050) associated with MDD, highlighting its genetic complexity. The present study helps explain the complex interactions between HHV-6 infection, genetics and MDD onset, improving understanding of how SNPs in JARID2 contribute to HHV-6 infection and MDD onset; these findings may impact future approaches to diagnosing and treating MDD.

## Introduction

Major depressive disorder (MDD) is a global health concern that can lead to suicide. The World Health Organization estimates that 350 million individuals worldwide have MDD (1). MDD is common in Thailand and its incidence varies by age group; studies have reported rates of 7.0-21.4% in individuals aged 19-22 years, 12.0% in individuals >30 years, 39.10% in those >40 years, 9.8-29.2% in those >45 years and 6.5-18.5% in those >47 years (2-7). Its etiology is not entirely known. Many factors are associated with MDD, including viral infection that causes inflammation in the brain, abnormal hormone, neurotransmitter and proinflammatory cytokine levels and genetic factors (8-10).

Acute and latent infection can affect behavior and mental health. Increased proinflammatory cytokine levels and decreased serotonin and norepinephrine levels cause an inflammatory reaction in response to viral infection of microglia, oligodendrocytes and astrocytes (11,12). Previous research has revealed that elevated proinflammatory cytokine levels are associated with MDD and infection by the varicella zoster, Epstein-Barr, cytomegalovirus, Borna disease and herpes simplex viruses (12-16). Human herpes virus 6 (HHV-6) is a β-HV subfamily of HV family that can lead to chromatin modification and DNA methylation in target cells (17). Several studies have identified HHV-6 as a risk factor for MDD (16,18-21).

MDD is a complex mental disorder influenced by environmental, genetic and epigenetic factors. Genome-wide association studies (GWASs) have identified 178 genetic risk loci and >200 candidate genes (22-24). There is an association between epigenetic/genetic changes and aberrant neuromediators (serotonin, norepinephrine and dopamine) that can lead to MDD (1,25-27). These effects are hypothesized to be mediated by the metabolic disturbance of brain-derived neurotrophic factor (BDNF) in nerve tissue. The single nucleotide polymorphism (SNP) rs6265 in BDNF changes a valine to a methionine at codon 66 (Val66Met), which decreases activity-dependent release of BDNF and is associated with low BDNF levels and MDD pathogenesis (28-30). The dopaminergic system, including dopamine-producing cells, receptors and transporters, may serve a key role in MDD. Solute carrier family 6 member 3 (SLC6A3) regulates dopamine levels in the brain by inhibiting synaptic activity and inducing dopamine reuptake into presynaptic neurons (31-35). In addition, jumonji and AT-rich interaction domain containing 2 (JARID2) is a DNA-binding protein that regulates gene expression by modifying chromatin. SNP rs9383046 in JARID2 has been identified as a potential candidate gene for schizophrenia (36-38).

The interaction between several variables, including viral infection and genetic characteristics, may serve an important role in MDD onset. The present study investigated the association between HHV-6 infection and specific genetic factors [SLC6A3 (g.1394961C>T, rs40184), BDNF (g.27658368C>T, rs6265) and JARID2 (g.15281336G>A, rs9383046)] to elucidate their role in MDD onset. High-resolution melt analysis (HRM) for genetic analysis was performed, along with HHV-6 infection data and viral load assessment from a previous study (39). SNPs were assessed using whole-exome sequencing.

## Materials and methods

### Specimens

The present study, approved by the Committee on Human Research Ethics (approval nos. UP-HEC 1.3/013/65 and UBU-REC-68/2567), analyzed 471 buccal cell samples from a previous study (39). The MDD samples were collected using mail-in submissions following social media advertising in Thailand. A total of 376 female and 95 male participants were enrolled between July 2022 and June 2023. The participants included 360 healthy individuals, 59 MDD patients, 36 blood relatives of MDD patients, and 16 non-blood relatives of MDD patients. All participants' HHV-6 status was confirmed with quantitative PCR. The study specifically targeted patients aged 18-45 years, diagnosed based on the Diagnostic and Statistical Manual of Mental Disorders, Fifth Edition (40). Both recurrent MDD and first-episode cases were included. Certified hospital psychiatrists confirmed diagnoses using the Patient Health Questionnaire-9 (PHQ-9), with a score of ≥9 indicating MDD (39). Patients with MDD exhibited various forms of MDD, including recurrent episodes, acute phase and persistent MDD. Notably, patients with MDD did not present with comorbid conditions such as diabetes, hypertension, chronic obstructive pulmonary disease or coronary artery disease. Family members of patients with MDD were recruited to determine potential risk factors for the condition. Healthy controls were individuals with no present or lifetime psychiatric problems, PHQ-9 score <9 and no congenital disease (39). First-degree relatives of individuals with suicidal ideation or MDD diagnosis were excluded from the control group.

### DNA extraction

DNA was extracted from buccal cells using the Genomic DNA Isolation kit (cat. no. PDC11-0100; Bio-Helix Co.) following the manufacturer's protocol as previously described (39).

### HHV-6 DNA detection by quantitative (q)PCR and viral load

qPCR-based HHV-6 DNA (U97 gene) status and viral load data were taken from a previous study (39).

### SNP detection by HRM

SNPs in SLC6A3 (g.1394961C>T, rs40184), JARID2 (g.15281336G>A, rs9383046) and BDNF (g.27658368C>T, rs6265) were detected by HRM analysis with amplicon sizes of 100, 88 and 146 bp, respectively. Table I shows primer sequences used for HRM analysis. PCR was performed in duplicate using the 5X FiREPOL Eva Green HRM Mix Plus (Solis Bio Dyne) as previously described (39,41,42). Positive controls for SLC6A3 (C/T, rs40184), JARID2 (G/A, rs9383046), and BDNF (C/T, rs6265) were utilized following sequencing confirmation. The primers used for HRM analysis are listed in Table I.

### DNA sequencing

Sanger sequencing was performed to confirm the SNPs in SLC6A3 (g.1394961C>T, rs40184), JARID2 (g.15281336G>A, rs9383046), and BDNF (g.27658368C>T, rs6265). A total of 10 samples were analyzed for BDNF, 45 for SLC6A3, and 15 for JARID2, randomly selected from different patterns of HRM*.* The primers used for sequencing are listed in Table I. The sequences were analyzed by comparison with GenBank reference sequences, BDNF (accession no. NC_000011.10:27658340-27658399) on *Homo sapiens* chromosome 11, DAT1 (accession no. NC_000005.10:1394924-1394997) on chromosome 5 and JARID (accession no. NC_000006.12:15281287-15281391) on chromosome 6] using BioEdit version 7.2; bioedit.software.informer.com/7.2/).

### Whole-exome sequencing

Whole-exome sequencing data to identify SNPs in samples taken from patients with MDD, blood relatives and the healthy controls was obtained from a previous study (39).

### Statistical analysis

Data were analyzed using IBM SPSS software (Version 16.0, SPSS Inc.)*.* Categorical variables were compared using Pearson's χ^2^ test. Continuous variables, reported as the mean ± standard deviation of ≥2 independent experimental repeats were compared between two groups using independent Student's or unpaired t-test and between more >2 groups using a one-way analysis of variance followed by LSD test or Median or Mann-Whitney U test. P<0.05 was considered to indicate a statistically significant difference.

## Results

### Association between SLC6A3 (g.1394961C>T, rs40184), BDNF (g.27658368C>T, rs6265), and JARID2 (g.15281336G>A, rs9383046) SNPs with MDD

SNPs were detected in healthy controls, patients with MDD and their blood and non-blood relatives using HRM analysis (Tables II and III). The variant T allele of rs40184 in SLC6A3 was significantly more common in patients with MDD (18/40, 45%) than in healthy controls [29/231, 12.6%; odds ratio (OR), 5.699, 95% confidence interval (CI), 2.734-11.879. Similarly, the variant T allele of rs6265 in BDNF was significantly more common in patients with MDD (30/54, 55.56%) than in healthy controls (117/292, 40.06%; OR, 1.870, 95% CI, 1.041-3.358). However, the frequency of variant A allele of rs9383046 in JARID2 did not differ significantly between patients with MDD and healthy controls.

**Figure 1:**
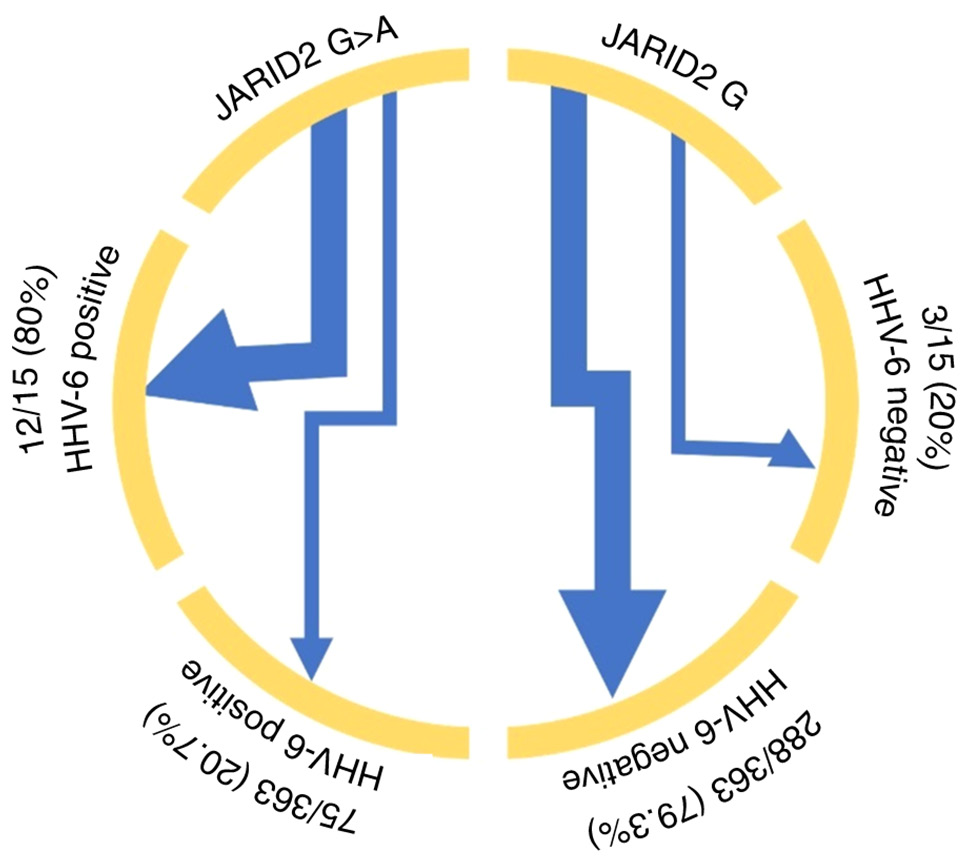
Association between HHV-6 infection and JARID2 rs9383046. HHV-6, human herpes virus 6; JARID2, jumonji and AT-rich interaction domain containing 2.

HHV-6 infection status was associated with SNP rs9383046 (G>A) in JARID2 but not with SNP rs40184 (C>T) in SLC6A3 or SNP rs6265 (C>T) in BDNF (Fig. 1; Table IV). The A allele of SNP rs9383046 in JARID2 was significantly more common in individuals with HHV-6 infection (80.0%, or 12 out of 15) than in those with the wild-type G allele (75/363, 20.7%; OR, 15.360, 95% CI, 4.226-55.823). This suggests that SNP rs9383046 (G>A) in JARID2 may be associated with HHV-6 infection.

### High HHV-6 viral load associated with JARID2 SNP (g.15281336G>A)

**Figure 2:**
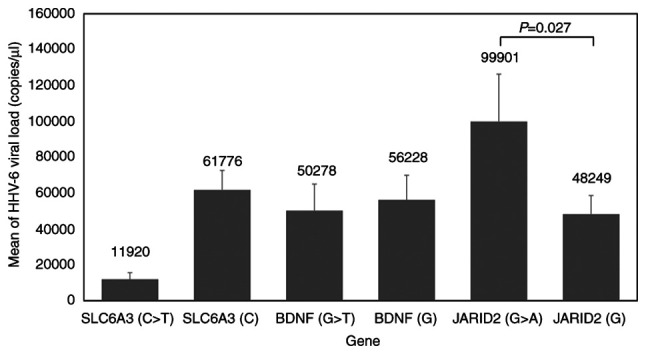
HHV-6 viral load in individuals with polymorphism of SLC6A3, BDNF and JARID2. HHV-6, Human herpes virus 6; SLC6A3, Solute carrier family 6 member 3; BDNF, brain-derived neurotrophic factor; and JARID2, jumonji and AT-rich interaction domain containing 2.

Our previous study used qPCR to determine viral load in HHV-6-positive cases (39). HHV-6 loads were significantly higher in individuals with the G>A genotype (99,990.85±118,392.64) than in those with the G genotype (48,249.30*±*101,216.28 copies/ng DNA) for SNP rs9383046 in JARID2 (Fig. 2).

### JARID2 SNP in MDD

**Figure 3:**
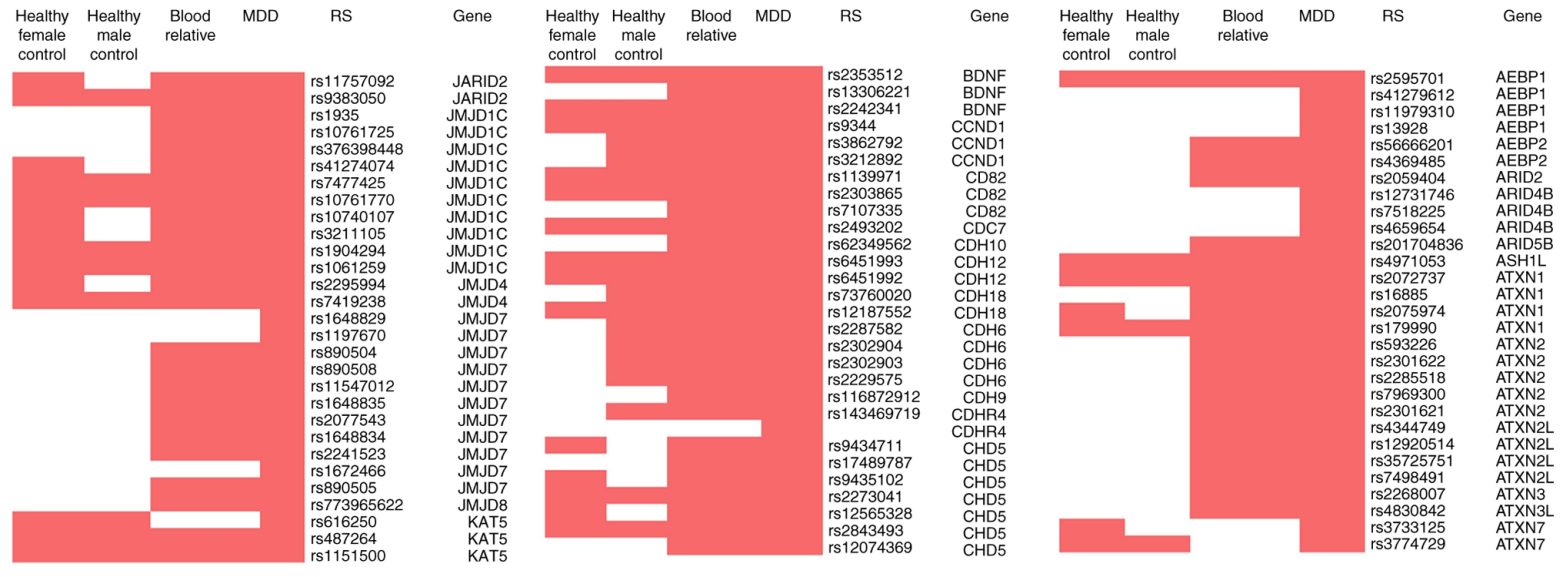
Association between single nucleotide polymorphisms in *JARID2* and associated pathways. JARID2, jumonji and AT-rich interaction domain containing, 2; MDD, major depressive disorder; RS, Reference SNP.

Whole-exome sequencing was used to analyze the point mutation status of samples from four participants: Patient with MDD, their first-degree relative and a healthy female and male, all aged between 21 and 30 years. The sample was randomly selected from patients with MDD who had a first-degree (healthy) relative in the same age group. The whole-exome sequencing results are shown in Fig. 3, revealing 484 SNPs in JARID2 in MDD. Previous literature reviews have reported that the following genes are associated with JARID2 and its associated pathways: AE Binding Protein 2 (AEBP2), AT-rich interaction domain (ARID), AT-rich sequences (AT-rich), ataxin 1 (ATXN1), brain-derived neurotrophic factor (BDNF), cluster of differentiation 82 (CD82), cadherin 13 (CDH13), cyclin D1 (CCND1), D-Amino acid oxidase activator (DAOA), differentially methylated regions (DMRs), dystrobrevin-binding protein 1 (DTNBP1), enhancer of zeste 2 polycomb repressive complex 2 subunit (EZH2), histone 3 lysine 27 (H3K27), histone methyltransferases, homeobox A cluster (HOXA), IFN-γ), isoflavone reductase (IFR), IL-18), IL-6), Jumonji domain-containing protein-3 (JMJD3), lysine acetyltransferase 5 (KAT5), Lysine Demethylase 2B (KDM2B), Neuregulin 1 (NRG1), PHD finger protein 1 (PHF1), polycomb repressive complex 2 (PRC2), RB Binding Protein 4 (RBBP4), RB transcriptional corepressor like 2 (RBL2), regulator of G protein signaling 4 (RGS4), Sirtuin (SIRT5), SLC6A3, SMAD family member 2 (SMAD2), *SOX, STAT3*, transcription factor AP-2α (FAP2A), transforming growth factor beta (TGF-ß), Vascular endothelial growth factor (VEGF) and zinc finger E-box binding homeobox 1 (ZEB) (36,37,42-44). There was a total of two SNPs each in JARID2 (rs11757092 and rs9383050) and JMJD4 (rs2295994 and rs7419238) and three SNPs in BDNF (rs2242341, rs2353512, and rs10835210; Table V).

## Discussion

The present study aimed to identify genetic factors associated with MDD. SLC6A3, BDNF and JARID2 were chosen because of their potential involvement in MDD pathways and previous research findings (28,30,31,34,36,38). To the best of our knowledge, the present study is the first to report on JARID2 and BDNF variations between patients with MDD and healthy individuals in Thailand. The present findings revealed an association between the SNP rs40184 in SLC6A3 and MDD, aligning with the results of other studies (34,45). This SNP alters SLC6A3 expression, which is associated with dopamine levels in the brain and depression severity (46,47). Previous studies have reported associations between dopamine transporter (DAT) levels and various psychiatric disorders including MDD and bipolar disorder (33,45,48). However, further investigation is necessary to elucidate these relationships. To comprehend the role of SLC6A3 (which encodes DAT) in psychiatric conditions, it must be explored within the broader context of phenotypical and allelic heterogeneity, as well as gene-environment interactions. Various SNPs in BDNF have been associated with MDD and suicide, including rs12273363, rs7124442, rs10767664, rs962369, rs908867 and especially rs6265 (Val66Met) (49-54). The rs6265 SNP decreases the activity-dependent release of BDNF (55). Furthermore, a previous study indicated an association between low BDNF levels and MDD pathogenesis (30).

To date, 500,000-1,000,000 SNPs have been identified in individuals with MDD compared with healthy controls. However, GWASs have not definitively demonstrated the influence of these SNPs on underlying mechanisms of mental disorders. These SNPs typically exhibit minimal phenotypic effects, with ORs ranging from 1.0 to 1.2 (9,56-59). Despite the subtle effects of individual SNPs, twin studies have revealed a significant genetic component in MDD (60,61). For example, one study found 37% heritability for recurrent unipolar MDD, with only minor environmental risk factors (62). More broadly, various studies have estimated the heritability of MDD to be between 30 and 50% (21,24). SNPs and other factors appear to be weakly associated with MDD. However, genetic variation varies between population, genetic background, and sequencing technology. The genetic mechanism for heritability remains unclear. Viruses or other factors may contribute to MDD pathophysiology (8,10,59). HHV-6 is often transmitted from mother to child and within families and can persist for life, this genetic polymorphism in HHV-6 may impact the heritability of MDD (21).

The present study identified a novel association between HHV-6 status and an SNP in JARID2. To the best of our knowledge, the present study is the first to explore the relationship between JARID2 and HHV-6, including both infection status and viral load, in the context of MDD. Variant A allele of SNP rs9383046 in JARID2 was more prevalent among individuals positive for HHV-6 infection and was positively associated with viral load. Previous research has linked pathogenic polymorphisms in JARID2 to a neurodevelopmental syndrome marked by developmental delays, cognitive impairment, hypotonia, autism and behavioral abnormalities (39,63). Additionally, rs326221458 SNP has been shown to affect JARID2 expression, which influences aggressive behavior in weaned pigs (64).

A total of six main hypotheses have been proposed to explain MDD pathogenesis: Hypothalamic/pituitary/adrenal axis dysfunction, monoamine imbalance, inflammation, genetic and epigenetic anomalies, structural and functional brain remodeling and social psychological factors. However, no single hypothesis can fully explain the pathology of MDD (8,10). The present study did not identify a direct association between MDD and JARID2. However, JARID2 may be directly associated with HHV-6 infection and indirectly with neurodevelopmental disorders, potentially increasing the risk of developing MDD. JARID2 polymorphisms may increase HHV-6 viral load, which is associated with inflammation and may contribute to MDD development (22,40). JARID2 serves as a transcriptional repressor protein and regulates histone methyltransferase complexes (65). Viral infections alter the expression of lysine demethylase 2B (KDM2B/NDY1), enhancer of zeste 2 polycomb repressive complex 2 subunit (EZH2) and JARID2. This alteration actively degrades growth factor-independent 1 transcriptional repressor (GFI1), thereby promoting activation of the major immediate early promoter (MIEP) of human cytomegalovirus (HCMV). The combination of KDM2B/NDY1, EZH2 and JARID2 promotes histone H3 K27 trimethylation, which is associated with transcriptional suppression. EZH2, responsible for histone H3 K27 trimethylation, interacts with JARID2, a non-canonical jumonji domain protein that modulates EZH2 methyltransferase activity. HCMV infects cells by targeting GFI1 through various mechanisms. GFI1 mRNA and protein levels are rapidly downregulated upon viral exposure, likely via degradation (18,66-68). HHV-6 is similarly activated, with GFI1 rapidly degrading after HHV-6 infection, activating the MIEP.

Future research should further examine the roles of KDM2B/NDY1, EZH2, JARID2 and JMJD3 using the SNPs in JMJD1, JMJD4, JMJD7, JMJD8, and JARID2 identified in the present study (18,66-68). Moreover, a common feature of HHV is the association of latent genomes with polycomb group proteins, specifically PRC2.2, which includes JARID2 and is associated with H3K27me3 (43,69).

Several mechanisms have been proposed for MDD onset, including monoamine theory, neuroendocrine mechanisms, neurotrophic hypothesis and neuroinflammation (8,10). JARID2 may influence HHV-6 and tumor necrosis factor-alpha (TNF-α)-induced inflammation through various indirect pathways. Neuroinflammation and cytokines are connected to monoamine deficiency, suppressed neurogenesis, and dysregulation of the hypothalamic/pituitary/adrenal axis. Chronic stress and inflammation can cause peripheral inflammatory markers to cross the blood-brain barrier into the central nervous system, activating microglia (70-73). TNF-α promoter SNP (G>A) and elevated HHV-6 viral load, including factors like JARID2, lead to TNF-α overexpression and subsequent inflammation (39). Therefore, the current data suggest that HHV-6 may enhance viral load by promoting neuroinflammation through JARID2.

The present study had limitations. The primary limitation was small sample size. Another was the difference in mean ages between the MDD group and the healthy control group, which may cause bias in the results. The present study investigated SLC6A3 as a biomarker for MDD diagnosis, as well as JARID2 and HHV-6 as potential therapeutic targets. The role of JARID2 in MDD should be assessed by investigating its expression patterns.

In conclusion, the present study demonstrated significant association between SNPs in BDNF (rs6265) and SLC6A3 (rs28363170) and MDD. A higher HHV-6 viral load was found in participants carrying variant A allele of SNP rs9383046 (G>A) in JARID2. Whole-exome sequencing identified SNPs in JARID2 in patients with MDD, indicating interactions between HHV-6 infection, SNPs and MDD development.

## Figures and Tables

**Table I:** Primers used for HRM analysis and sequencing.

Gene	Sequence, 5'→3'	Size, bp	(Refs.)
BDNF (rs6265)	Forward: CTTGACATCATTGGCTGACACT	146	(41)
	Reverse: GCTCCAAAGGCACTTGACTACT		
DAT1 (rs40184)	Forward: CACAGTCTCGCGGCTTTT	100	(41)
	Reverse: TGGACCAACACACCCTTGA		
JARID2 (rs9383046)	Forward: ACTGGCTGTGTCTCACTCTT	88	(42)
	Reverse: TATTCACGTTCTTTTGCTCTTGGA		
JARID2 (rs9383046)^a^	Forward: ATTTGACCCACACTGGCTGT	96	NA
	Reverse: TCACGTTCTTTTGCTCTTGGAC		

^a^Used for sequencing; the same *DAT1* and *BDNF* primers were used for both HRM analysis and sequencing. BDNF, brain-derived neurotrophic factor; DAT1, dopamine transporter 1; JARID2, jumonji and AT-rich interaction domain containing 2; HRM, high resolution melt; NA, not applicable.

**Table II:** Allele distribution.

Gene	Allele	n	Healthy controls (%)	Patients with MDD (%)	Blood relatives (%)	Non-blood relatives (%)	P-value
*DAT1*	T	52	29 (12.6)	18 (45.0)	4 (15.4)	1 (7.7)	<0.001
	C	258	202 (87.4)	22 (55.0)	22 (84.6)	12 (92.3)	
*BDNF*	T	161	117 (40.1)	30 (55.6)	8 (40.0)	6 (50.0)	0.034
	C	217	175 (59.9)	24 (44.4)	12 (60.0)	6 (50.0)	
*JARID2*	A	15	14 (4.7)	0 (0.0)	1 (4.2)	0 (0)	0.417
	G	368	283 (95.3)	47 (100.0)	23 (95.8)	11(100)	

DAT1, dopamine transporter 1; BDNF, brain-derived neurotrophic factor; JARID2, jumonji and AT-rich interaction domain containing 2; MDD, major depressive disorder; N, numbers.

**Table III:** Allele distribution in patients with MDD and the healthy controls.

Gene	Group	n	T (%)	C (%)	P-value	OR (95% CI)
*DAT1*	MDD	40	18(45)	22(55)	<0.001	5.699
	Healthy	231	29 (12.55)	202 (87.45)		(2.734-11.879)
*BDNF*	MDD	54	30 (55.56)	24 (44.44)	0.034	1.870
	Healthy	292	117 (40.06)	175 (59.93)		(1.041-3.358)
			A	G		
*JARID2*	MDD	47	0 (0)	47(100)	0.129	NA
	Healthy	297	14 (4.71)	283 (95.29)		

DAT1, dopamine transporter 1; BDNF, brain-derived neurotrophic factor; JARID2, jumonji and AT-rich interaction domain containing 2; MDD, major depressive disorder; NA, not applicable.

**Table IV:** Association between HHV-6 status and single nucleotide polymorphism alleles.

			HHV-6 status		
Gene	Allele	n	Positive (%)	Negative (%)	P-value
*DAT1*	T	51	15 (29.4)	36 (70.6)	0.916
	C	258	74 (28.7)	184 (71.3)	
*BDNF*	T	161	36 (22.4)	125 (77.6)	0.578
	C	215	43 (20.0)	172 (80.0)	
*JARID2*	A	15	12 (80.0)	3 (20.0)	<0.001
	G	363	75 (20.7)	288 (79.3)	

DAT1, dopamine transporter 1; BDNF, brain-derived neurotrophic factor; JARID2, jumonji and AT-rich interaction domain containing 2.

**Table V:** Association of single nucleotide polymorphisms in *JARID2* and genes in associated pathways.

Reference allele	Alternative allele	Gene	RS	Target gene
T	C	AEBP2	rs56666201	AE Binding Protein 2
A	G	AEBP2	rs4369485	
A	G	AEBP1	rs2595701	
T	A	AEBP1	rs11979310	
G	A	AEBP1	rs41279612	
A	G	AEBP1	rs13928	
A	G	ARID4B	rs4659654	A-T Rich Interaction Domain
T	C	ARID4B	rs12731746	
A	C	ARID4B	rs7518225	
T	C	ARID4B	rs139681730	
G	A	ARID5B	rs201704836	
G	A	ARID2	rs2059404	
C	G	ATXN2	rs2301622	Ataxin 1
C	T	ATXN2	rs2301621	
A	G	ATXN2	rs2285518	
C	T	ATXN2	rs7969300	
A	G	ATXN2	rs593226	
T	G	ATXN3	rs3092822	
G	T	ATXN3	rs7158733	
G	A	ATXN3	rs761552	
C	T	ATXN3	rs1048755	
T	C	ATXN3	rs2268007	
T	C	ATXN3	rs8003041	
G	C	ATXN3	rs4904834	
G	A	ATXN2L	rs12920514	
A	G	ATXN2L	rs4344749	
T	A	ATXN2L	rs7498491	
C	T	ATXN2L	rs35725751	
C	T	ATXN7	rs117130898	
C	T	ATXN7	rs3733125	
G	A	ATXN7	rs3774729	
G	A	ATXN1	rs16885	
G	A	ATXN1	rs2072737	
T	C	ATXN1	rs2075974	
A	G	ATXN1	rs179990	
G	A	ATXN7L1	rs940370	
C	T	ATXN3L	rs4830842	
G	C	BDNF-AS	rs2242341	Brain-derived neurotrophic factor
T	C	BDNF	rs2353512	
C	A	BDNF-AS	rs10835210	
C	T	BDNF	rs13306221	
G	A	CD82	rs2303865	CD82
A	G	CD82	rs1139971	
C	G	CD82	rs7107335	
A	G	CDHR4	NA	Cadherin-13
T	A	CDHR4	rs143469719	
G	A	CDHR4	rs76282326	
G	A	PCDH7	rs28481709	
G	A	PCDH7	rs28387015	
G	A	PCDH7	rs73216844	
G	A	PCDH7	rs1047012	
G	A	PCDH7	rs977931	
G	C	PCDH7	rs4580617	
A	G	PCDH7	rs10006845	
T	G	PCDH7	rs16867990	
G	A	PCDH18	rs144391842	
T	C	PCDH18	rs151070417	
A	G	PCDH18	rs10018837	
C	G	PCDH18	rs10006580	
G	A	PCDH18	rs28566714	
C	T	CDH18	rs73760020	
T	C	CDH18	rs12187552	
A	G	CDH12	rs6451992	
A	G	CDH12	rs6451993	
C	T	CDH10	rs62349562	
C	T	CDH9	rs116872912	
C	T	CDH6	rs2287582	
C	T	CDH6	rs2302904	
A	G	CDH6	rs2302903	
T	C	CDH6	rs2229575	
C	T	CCND1	rs3862792	Cyclin D1
G	A	CCND1	rs9344	
A	G	CCND1	rs3212892	
G	A	DAOA	rs2391191	D-amino acid oxidase activator
T	C	DMRTC2	rs2305809	Differentially methylated region MRs
G	C	DMRT1	rs279895	
T	C	DMRT3	rs6477419	
G	A	DMRT2	rs202027041	
G	C	DMRT2	rs3824419	
C	G	DMRTA1	rs201444816	
C	T	DMRTC1B	rs201167658	
C	T	DTNBP1	rs4236167	Dystrobrevin binding protein 1
G	T	DTNBP1	rs6926401	
T	C	DTNBP1	rs7758659	
A	G	DBNDD2	rs1127497	
A	G	EZH2	rs740949	Enhancer of zeste 2 polycomb repressive complex 2
A	C	EZH2	rs41277434	
A	G	EZH2	rs2072407	
C	G	EZH2	rs2302427	
A	G	EZH2	rs10274535	
T	C	CHD5	rs6696489	Methylation of histone H3
C	A	CHD5	rs59788818	
A	G	CHD5	rs2843493	
A	G	CHD5	rs2273041	
G	A	CHD5	rs55930553	
G	C	CHD5	rs2273033	
C	T	CHD5	rs2235790	
T	G	CHD5	rs3765452	
A	G	CHD5	rs2250358	
A	G	CHD5	rs2746066	
T	A	CHD5	rs2785582	
A	C	CHD5	NA	
C	G	CHD5	rs17489787	
A	G	CHD5	rs9435102	
T	C	CHD5	rs12565328	
G	A	CHD5	rs9434711	
C	G	CHD5	rs12074369	
C	T	PRMT6	rs2232016	Histone methyltransferase
T	C	ASH1L	rs4971053	
A	C	ASH1L	rs10908466	
G	A	RBBP5	rs11240356	
G	A	RBBP5	rs7515178	
G	A	SMYD2	rs6540819	
G	A	SMYD2	rs1134647	
T	G	SMYD2	rs2291830	
T	C	SMYD2	rs2270704	
C	T	SMYD2	rs1874804	
G	A	SETD2	rs2290547	
G	A	SETD2	rs4082155	
A	G	SETD2	rs6767907	
C	G	SETD2	rs6442059	
A	G	NSD2	rs489550	
G	C	HOXA1	NA	Homeobox A cluster
G	A	HOXA1	rs778380747	
T	C	HOXA1	NA	
T	G	HOXA1	NA	
T	C	HOXA1	NA	
C	T	HOXA1	NA	
C	T	HOXA1	rs10951154	
G	A	HOXA1	rs577426612	
T	C	HOXA4	rs17449108	
A	C	HOXA4	rs6957209	
A	G	HOXA-AS3	rs62454420	
A	C	HOXA7	rs2301720	
C	T	HOXA7	rs2301721	
A	G	HOXA9	NA	
A	G	IFNLR1	rs946671	Interferon-γ
T	C	IFNLR1	rs4649195	
T	C	IFNLR1	rs7552000	
T	C	IFNAR2	rs2834158	
A	G	IFNAR2	rs2252639	
C	T	IFNAR2	rs9984273	
A	G	IFNGR2	rs9808753	
C	G	IFNGR2	rs11910627	
T	C	IFNGR2	rs1532	
A	G	IFNGR1	rs1887415	
A	C	IFNGR1	rs11914	
G	A	IFNGR1	rs1327475	
C	G	IFNGR1	rs9376269	
A	G	IFNGR1	rs2234711	
G	A	IFNB1	rs1051922	
A	G	IFNA21	rs2939	
A	G	IFNA4	rs3750479	
T	A	IFNA4	rs3750480	
C	T	IFNA4	rs1062571	
T	C	IFNA7	rs76644201	
G	T	IFNA10	rs56035072	
T	C	IFNA16	rs3919593	
A	C	IFNA17	rs9298814	
A	G	IFNA17	rs7025879	
G	T	IFNA17	rs10117962	
G	A	IFNA5	rs10757212	
G	A	IFNA6	rs2988573	
A	G	IFNK	rs700785	
G	A	IFRD2	rs2229647	Isoflavone reductase
A	G	IFRD2	rs2071205	
A	G	IFRD2	rs1076872	
C	T	IFRD1	rs55884191	
T	C	IFRD1	rs34349457	
C	T	IFRD1	rs6968084	
T	G	IFRD1	rs2253962	
G	A	IFRD1	rs2074796	
A	C	IL18BP	rs2298455	Interleukin-18
C	T	IL18R1	rs1035130	
A	G	IL18R1	rs4851570	
A	G	IL18RAP	rs1558651	
G	A	IL18RAP	rs6708413	
G	A	IL6R; SHE	rs11265621	Interleukin-6
A	G	IL6ST	rs4865999	
T	A	JARID2	rs11757092	Jumonji and AT rich interactive domain 2
A	G	JARID2	rs9383050	
C	T	JARID2	NA	
A	C	JMJD4	rs2295994	Jumonji domain-containing protein-3
G	A	JMJD4	rs7419238	
C	G	JMJD1C	rs1935	
G	A	JMJD1C	rs376398448	
T	A	JMJD1C	rs10740107	
G	A	JMJD1C	rs3211105	
A	T	JMJD1C	rs1904294	
G	C	JMJD1C	rs41274074	
A	T	JMJD1C	rs10761725	
T	C	JMJD1C	rs7477425	
G	C	JMJD1C-AS1	rs1061259	
A	G	JMJD1C-AS1	rs10761770	
G	C	JMJD7	rs2241523	
C	G	JMJD7	rs2303516	
G	A	JMJD7	rs890505	
T	C	JMJD7	rs890504	
C	G	JMJD7	rs11547012	
C	G	JMJD7	rs2077543	
A	C	JMJD7	rs1648835	
G	C	JMJD7	rs1648834	
C	T	JMJD7	rs1672466	
G	A	JMJD7	rs1648829	
A	G	JMJD7	rs1197670	
G	A	JMJD7	rs890508	
C	T	JMJD8	rs767035682	
A	G	JMJD8	NA	
A	G	JMJD8	NA	
G	A	JMJD8	rs773965622	
G	T	KAT5	rs2236682	Lysine acetyltransferase
A	G	KAT5	rs616250	
T	C	KAT5	rs1151500	
T	G	KAT5	rs487264	
A	G	KATNAL1	rs202087	
T	C	KATNBL1	rs74482733	
A	G	KAT8	rs1549295	
A	G	KAT8	rs1549294	
C	T	KAT8	rs1549293	
A	G	KATNB1	rs2967152	
C	G	KATNB1	rs2967153	
C	T	KATNB1	rs9938236	Katanin regulatory subunit B1-like 1
C	T	KATNB1	rs12922275	
G	A	KATNB1	rs76370907	
C	T	KDM1A	rs967605	Lysine demethylase 2B
A	C	KDM1A	rs2072945	
T	C	KDM1A	rs2072944	
C	A	KDM4A	rs586339	
G	A	KDM5B	rs4310498	
G	C	KDM5B	rs1141109	
G	A	KDM5B	rs1141108	
C	T	KDM5B	rs3196669	
G	A	KDM5B	rs12028388	
T	C	KDM2A	rs3741189	
A	G	KDM4D	rs76057256	
G	A	KDM4D	rs3740853	
G	A	KDM4E	rs2020210	
A	G	KDM4E	rs10752685	
A	G	KDM4E	rs28412010	
A	G	KDM5A	NA	
T	G	KDM5A	NA	
A	G	KDM5A	NA	
T	C	KDM5A	rs771421847	
G	A	KDM5A	rs2229351	
A	G	KDM5A	rs11062385	
C	T	KDM5A	rs4980885	
A	T	KDM5A	rs7965303	
G	A	KDM2B	rs12427382	
A	C	KDM2B	rs11065575	
C	T	KDM2B	rs1064951	
G	C	KDM2B	rs2288154	
G	A	KDM2B	rs3751131	
G	A	KDM2B	rs10849885	
T	G	KDM2B	rs28461264	
G	A	KDM8	rs877585	
A	C	KDM8	rs908382	
T	G	KDM8	rs11645703	
A	G	KDM6B	rs80152199	
C	T	KDM6B	rs2270516	
T	C	KDM6B	rs2270517	
C	A	KDM6B	rs3744247	
C	T	KDM6B	rs3744248	
C	A	KDM6B	rs3736306	
T	C	KDM4B	rs2240678	
T	G	KDM4B	rs1017820	
C	T	KDM4B	rs148048943	
A	G	KDM4B	rs2620836	
G	A	KDM4B	rs2613786	
A	G	KDM3A	rs2030259	
G	A	KDM3A	rs12714187	
G	A	KDM3A	rs61748134	
T	C	KDM3A	rs4832290	
A	G	KDM3A	rs75816635	
C	T	KDM3B	rs4835678	
T	G	KDM3B	rs12522867	
G	A	KDM3B	rs10073922	
G	A	KDM3B	rs6865472	
A	G	KDM3B	rs2269951	
T	C	KDM3B	rs7726234	
A	G	KDM3B	rs192834842	
C	T	KDM3B	rs17599026	
T	C	KDM1B	rs429158	
G	A	KDM1B	rs214596	
T	C	KDM1B	rs214585	
G	A	KDM7A	rs12703533	
G	T	KDM7A	rs6950119	
T	G	KDM7A	rs1062277	
C	G	KDM7A	rs59225858	
T	C	KDM4C	rs7040131	
G	A	KDM4C	rs10815499	
G	A	KDM4C	rs2296067	
A	G	KDM4C	rs35389625	
G	T	KDM4C	rs818883	
G	A	KDM4C	rs1570512	
C	T	KDM4C	rs3763651	
G	C	KDM4C	rs10758825	
C	G	KDM4C	rs1407863	Lysine demethylase 4C
C	A	KDM6A	rs6611055	
C	A	KDM6A	rs2230018	
G	A	KDM6A	rs20539	
T	C	KDM5C	NA	
T	C	KDM5C	NA	
A	G	KDM5C	rs1536247	
C	A	KDM5C	rs1977364	
C	G	MIR4711	NA	MicroRNA 4740
G	T	MIR4711	NA	
A	G	MIR4711	rs111566161	
C	A	MIR4707	rs2273626	
G	A	MIR4706	rs2296320	
A	G	MIR4708	rs12881755	
A	C	MIR4713HG	rs4646	
T	A	MIR4713HG	rs28757202	
G	A	MIR4713HG	rs28757201	
A	C	MIR4713HG	rs4324076	
T	C	MIR4713HG	rs3759811	
C	A	MIR4713HG	rs727479	
C	T	MIR4721	rs548287959	
C	G	MIR4719	rs34106659	
A	G	MIR4719	rs58353328	
T	C	MIR4719	rs7500280	
G	A	MIR4719	rs7499278	
G	C	MIR4752	rs4112253	
G	T	MIR4752	rs375002929	
T	A	MIR4752	rs3890103	
C	T	MIR4752	rs7247101	
A	C	MIR4752	rs7246998	
T	C	MIR4752	rs7248086	
T	C	MIR4752	rs7248089	
G	C	MIR4779	rs77373668	
T	C	MIR4771-1	rs866077379	
G	A	MIR4771-1	rs4591335	
G	A	MIR4771-1	rs200567153	
T	C	MIR4771-1	rs4047215	
T	G	MIR4771-1	rs2245238	
T	C	MIR4771-1	rs1522044	
C	A	MIR4776-1;-2	NA	
T	G	MIR4776-1;-2	NA	
A	C	MIR4799	NA	
G	A	MIR4799	NA	
G	A	NRG1	rs3924999	Neuregulin 1
C	T	NRG1	rs57944175	
C	A	NRG1	rs3735776	
G	A	SETDB2-PHF11	rs2077848	PHD finger protein 1
G	T	SETDB2-PHF11	rs7996852	
G	A	SETDB2-PHF11	rs2057413	
A	G	SETDB2-PHF11	rs11619265	
A	G	PHF11	rs2031532	
A	G	PHF11	rs3765526	
C	G	PHF11	rs2274277	
G	A	PHF11	rs1046295	
G	A	PHF1	rs3116713	
G	T	PHF1	rs3106196	
G	A	PHF10	rs12663375	
C	T	PHF10	rs7760142	
A	G	PHF10	rs3807063	
G	A	PHF14	rs218965	
G	A	PHF14	rs2301960	
C	A	PHF14	rs28394821	
T	A	PHF20L1	rs2244885	
C	T	PHF20L1	rs16904746	
C	T	PHF20L1	rs756201	
G	A	PHF24	rs2279790	
G	A	PHF24	rs2279791	
G	A	PHF2	rs10992812	
A	G	PHF2	rs3763605	
T	A	PHF2	rs10761251	
T	C	PHF2	rs10992836	
G	A	PHF2	rs3750359	
A	G	PHF2	NA	
C	T	PHF2	NA	
G	A	PHF2	rs374331864	
A	T	PHF2	NA	
T	G	PHF2	NA	
T	C	PHF2	NA	
G	C	PHF19	rs3753029	
A	G	PHF19	rs1056567	
G	C	PHF19	rs4836833	
T	C	PHC2	rs72658237	Polycomb repressive complex 2
C	A	PHC2	rs41265891	
A	G	PHC2	rs6425816	
T	C	PHC2	NA	
G	A	PHC2	NA	
G	A	PHC2	rs34710284	
T	C	RBBP4	rs2762904	RB binding protein 4
A	C	RBBP4	rs359955	
A	G	RBBP4	rs359956	
A	C	RBBP4	rs607202	
T	C	RBBP4	rs1320511	
C	T	RBBP4	rs55707864	
T	C	RBBP6	rs7195386	
T	C	RBBP8	rs2336916	
T	C	RBBP8	rs78439784	
A	G	RBBP9	rs3748450	
A	G	RBBP9	rs2424217	
A	G	RBBP9	rs2247746	
A	C	RBBP8NL	rs2236200	
A	C	RBBP7	rs5924532	
C	T	RBBP7	rs67984110	
C	T	REPS2	rs73189116	
T	A	REPS2	rs1946395	
A	G	RBL2	rs555878756	RB transcriptional corepressor like 2
G	A	RGS4	rs10917671	Regulator of G protein signaling 4
T	C	RGSL1	rs266508	
A	G	RGSL1	rs7535533	
C	A	RGSL1	rs266534	
G	C	RGSL1	rs6657620	
T	C	RGS16	rs1144566	
T	C	RGS16	rs509476	
T	C	RGS7	rs2275742	
T	A	RGS7	rs4659599	
G	C	RGS6	rs2238186	
C	A	RGS6	rs10131300	
T	G	RGS6	rs2239277	
C	G	RGS6	rs551152070	
A	C	RGS6	rs4617784	
A	G	RGS6	rs4346151	
A	G	RGS6	NA	
G	A	RGS6	NA	
A	G	RGS6	rs769852120	
A	G	RGS6	NA	
A	T	RGS6	rs11628539	
T	C	RGS11	rs57268939	
C	T	RGS11	rs74003728	
C	G	RGS11	rs117215141	
A	G	RGS9	rs2585858	
A	G	RGS9	rs9896245	
C	T	RGS9	rs12452285	
A	G	RGS9	rs2292592	
C	G	RGS9BP	rs3826926	
A	G	RGS12	rs2236052	
C	T	RGS12	rs10006362	
C	T	RGS12	rs118013842	
G	C	RGS12	rs3213507	
G	A	RGS12	rs374843920	
A	G	RGS12	rs2269497	
A	G	RGS7BP	rs889248	
T	A	RGS7BP	rs10939996	
T	A	RGS17	rs685449	
A	G	RGS22	rs6468700	
C	G	RGS22	rs3101322	
C	T	RGS22	rs922207	
G	A	RGS22	rs2980542	
A	G	RGS22	rs1471293	
T	C	RGS22	rs2453626	
T	C	RGS22	rs1460933	
C	T	RGS3	rs10981790	
A	T	RGS3	NA	
G	C	RGS3	rs12350531	
A	G	RGS3	rs12341266	
C	G	RGS3	rs10817493	
C	T	SIRT3	rs11246020	Sirtuin 5
C	A	SIRT3	rs11555236	
T	G	SIRT3	rs12365010	
C	T	SIRT3	rs34700713	
T	G	SIRT3	rs9795476	
A	G	SIRT7	rs1879568	
A	G	SIRT7	rs1879569	
A	G	SIRT6	rs350845	
T	G	SIRT6	rs7246235	
A	C	SIRT6	rs350843	
T	G	SIRT6	rs7260071	
C	T	SIRT6	rs352493	
G	C	SIRT2	rs45496398	
G	A	SIRT2	rs11879010	
G	A	SIRT2	rs11879029	
C	T	SIRT2	rs11667030	
A	G	SIRT2	rs11083483	
T	C	SIRT2	rs10410544	
G	A	SLC66A1	rs58790041	Solute carrier family 6
T	C	SLC6A17	rs7527375	
G	A	SLC6A17	rs12737742	
A	T	SLC6A17	rs1007692	
C	T	SLC6A17	rs12033312	
C	T	SLC6A5	rs76857783	
G	A	SLC6A5	rs1443547	
C	T	SLC6A5	rs7109418	
C	T	SLC6A5	rs2241941	
T	C	SLC6A5	rs1443548	
C	G	SLC6A5	rs1443549	
C	G	SLC6A5	rs72927519	
T	C	SLC6A5	rs2278649	
G	A	SLC6A5	rs1443551	
A	G	SLC6A5	rs7925597	
A	C	SLC6A5	rs7925624	
G	A	SLC6A5	rs11827415	
C	T	SLC6A5	rs4923548	
G	A	SLC6A5	rs2276433	
A	G	SLC6A12	rs646569	
T	C	SLC6A12	rs216248	
A	G	SLC6A12	rs216250	
C	T	SLC6A13	rs2289954	
C	T	SLC6A13	rs74057642	
C	G	SLC6A10P	rs395061	
C	T	SLC6A10P	rs3878528	
T	C	SLC6A10P	rs432506	
G	A	SLC6A10P	rs865876957	
A	G	SLC6A10P	rs796806794	
G	T	SLC6A10P	rs796465823	
C	T	SLC6A10P	rs542763347	
G	A	SLC6A10P	rs62046808	
C	T	SLC6A10P	rs28510469	
A	G	SLC6A2	rs5564	
T	G	SLC6A2	rs28613651	
T	C	SLC6A2	rs2279805	
G	A	SLC6A2	rs5569	
G	A	SLC6A2	rs998424	
C	T	SLC6A2	rs2242447	
G	T	SLC6A16	rs2278405	
G	A	SLC6A16	rs536601412	
C	T	SLC66A3	rs2271622	
T	C	SLC66A3	rs6432182	
T	C	SLC6A11	rs2304725	
T	C	SLC6A11	rs2272395	
C	T	SLC6A11	rs2272399	
C	T	SLC6A20	rs2251347	
A	G	SLC6A20	rs758386	
C	T	SLC12A7	rs6554618	
T	A	SLC12A7	rs116648138	
A	G	SLC6A19	NA	
G	A	SLC6A19	rs7732589	
A	G	SLC6A19	rs6554663	
A	G	SLC6A19	rs4975629	
T	C	SLC6A19	rs12513763	
G	A	SLC6A19	NA	
A	C	SLC6A19	NA	
G	A	SLC6A18	rs7704058	
T	A	SLC6A18	rs7728667	
C	T	SLC6A18	rs7705355	
T	C	SLC6A18	rs7728814	
A	G	SLC6A18	rs7724858	
G	C	SLC6A18	rs4975623	
C	A	SLC6A3	rs40358	
G	T	SLC6A3	rs460000	
C	T	SLC6A8	rs376038235	
C	A	SMAD9	rs3748305	SMAD family member 2
G	T	SMAD9	rs9576126	
T	C	SMAD9	rs182137303	
C	A	SMAD3	rs1866319	
A	G	SMAD3	rs1065080	
G	A	SMAD3	rs758586312	
C	T	SMAD5-AS1	rs3764942	
T	G	SMAD5-AS1	rs3764941	
G	T	SMAD5-AS1	rs548522421	
T	C	SMAD5-AS1	rs3764939	
A	G	SMAD5	rs4585442	
A	G	QSOX1	rs2298206	Sry-related HMG box
C	T	QSOX1	rs7521513	
C	T	QSOX1	rs1050154	
G	T	QSOX1	rs67343209	
T	C	QSOX1	rs7544147	
T	C	SOX8	rs237674	
C	T	SOX8	rs11542179	
A	C	SOX9	rs1042667	
C	T	SOX3	rs396467	
C	T	SOX13	rs2250538	
A	G	SOX13	rs7551756	
A	G	SOX6	rs4617548	
T	C	SOX6	rs7926424	
G	A	SOX6	rs12277740	
T	C	SOX5	rs4636755	
C	T	SOX5	rs7485662	
T	C	SOX5	rs7980561	
C	G	SOX21	rs1060474	
T	G	SOX21	rs2253604	
C	G	SOX14	rs1385306	
G	A	SOX30	rs35793864	
G	T	SOX30	rs12188040	
C	T	SOX30	rs777105078	
T	C	QSOX2	rs9696116	
T	C	QSOX2	rs10858247	
C	T	QSOX2	rs12684650	
C	A	QSOX2	NA	
T	C	QSOX2	rs12380852	
T	G	QSOX2	NA	
C	T	QSOX2	rs79849109	
T	C	QSOX2	rs567434269	
G	A	QSOX2	rs62579871	
C	T	SYNC	rs360042	Signal transducer and activator of transcription 3
G	A	TFAP2A	rs9367875	Transcription factor AP-2 alpha
A	G	TFAP2A	rs9370818	
C	T	TFAP2A	rs11968445	
A	G	TFAP2A	rs1925775	
G	A	TFAP2A	rs9477310	
C	T	TGFBR3	rs2493202	Transforming growth factor beta
A	G	TGFBR3	rs284878	
C	T	TGFBR3	rs1805112	
G	A	TGFBR3	rs11165441	
G	A	TGFBR3	rs1805110	
C	T	TGFBR3	rs1805109	
C	T	TGFB3	rs3917201	
A	G	TGFB1	rs1800469	
C	T	TGFBR1	rs11568753	
T	C	TGFBR1	NA	
A	G	TGFBR1	rs7041311	
G	A	TGFBR1	rs334354	
C	T	VEGFC	rs7664413	Vascular endothelial growth factor A
C	A	VEGFA	rs2146323	
G	A	VEGFA	rs3025009	
T	C	VEGFA	rs3025010	
C	T	YARS	rs144866833	
G	A	YARS	rs4951787	
A	G	ZEB1	rs189276857	Zinc finger E-box binding homeobox 1
G	A	ZEB1	rs220060	

RS, Reference single nucleotide polymorphism; NA, not applicable.

## Data Availability

The data generated in the present study may be found in the Sequence Read Archive database under accession number PRJNA1146872 or at the following URL: ncbi.nlm.nih.gov/sra/?term=PRJNA1146872.
